# SOUNET: Self-Organized Underwater Wireless Sensor Network

**DOI:** 10.3390/s17020283

**Published:** 2017-02-02

**Authors:** Hee-won Kim, Ho-Shin Cho

**Affiliations:** School of Electronics Engineering, Kyungpook National University, Daegu 41566, Korea; hwkim@ee.knu.ac.kr

**Keywords:** underwater wireless sensor networks, self-organization, self-healing, network connectivity, packet delivery ratio, node isolation, closed loop, underwater experiment

## Abstract

In this paper, we propose an underwater wireless sensor network (UWSN) named SOUNET where sensor nodes form and maintain a tree-topological network for data gathering in a self-organized manner. After network topology discovery via packet flooding, the sensor nodes consistently update their parent node to ensure the best connectivity by referring to the time-varying neighbor tables. Such a persistent and self-adaptive method leads to high network connectivity without any centralized control, even when sensor nodes are added or unexpectedly lost. Furthermore, malfunctions that frequently happen in self-organized networks such as node isolation and closed loop are resolved in a simple way. Simulation results show that SOUNET outperforms other conventional schemes in terms of network connectivity, packet delivery ratio (PDR), and energy consumption throughout the network. In addition, we performed an experiment at the Gyeongcheon Lake in Korea using commercial underwater modems to verify that SOUNET works well in a real environment.

## 1. Introduction

With the advancements in underwater communication technologies such as acoustic modems, hydrophones, and digital signal processing (DSP), underwater wireless sensor networks (UWSNs) have drawn considerable attention in various applications such as tactical surveillance, oceanographic observation, disaster prevention, undersea exploration, and assisted navigation [1,2,3]. In general, UWSNs consist of multiple fixed sensor nodes that monitor a specific area collaboratively and forward data to one or more data-gathering points known as the sink by multi-hop relaying or occasionally with the aid of mobile machines referred to as autonomous underwater vehicles (AUVs). Either the wireless communication links between the sensor nodes are preprogrammed prior to placement or a remote user can centrally assign them after placement. However, to configure the number of communication links manually, the centralized method requires substantial efforts. Furthermore, it is necessary to reconfigure the links whenever a sensor node is lost or added.

To eliminate such manual efforts required to maintain the network, *self-organizing network* (SON) technology is studied. In SON, individual sensor nodes discover network topology in a best-effort manner with local information only or sometimes via probabilistic approaches [4]. Along with the elimination of complexity, SON provides three strong points: *adaptability*, *robustness,* and *scalability*. Adaptability means that when a node is lost, the child nodes connected to the lost node as data senders try to find new parent nodes by themselves. Such an adaptive reconfiguration avoids unexpected data loss, which may occur for a long time or permanently in centralized networks. Additionally, a newly deployed sensor node is capable of joining the network using the limited information acquired through local observations. The active response to circumstances increases the network’s robustness in the sense that the network can handle the unexpected losses and additions of sensor nodes while maintaining the network’s performance up to a certain level. Lastly, scalability means that the network functions properly regardless of variations in the number of sensor nodes. However, the local decision-making cannot adequately meet the requirements for the global optimization of network performances because the sensor nodes are shortsighted without global information about the network. Thus, SONs have to be designed in such a way that the distributed local behaviors provide the closest optimum solution [5].

Clustering is an energy-efficient method to organize wireless sensor networks. For example, Lloret et al. proposed a clustering architecture to integrate different types of devices into several clusters [6]. Adjacent devices form a cluster group as cluster-members, which are connected to a cluster-head and to the same type of cluster-members in other clusters. Self-adaptive algorithms for newly added or lost nodes are also considered. However, the architecture is not for underwater environment. Furthermore, we consider homogeneous sensor nodes and non-clustered networks. Many previous works on SONs for UWSNs have also adopted a clustered architecture where the whole network is divided into several smaller groups for saving energy consumption during data gathering [7,8,9,10,11]. Huang et al. proposed a clustering head selection algorithm (CHSA) [9] that reduces overall energy consumption by organizing direction-sensitive clusters. A sensor node, which satisfies specific criteria—direction toward a sink node, is elected as the cluster-head. In addition, CHSA adopts a self-healing mechanism to recover link holes [12], which is also based on direction awareness. However, it is costly to set up separate devices such as the ultrashort baseline (USBL) system in each node. On the other hand, Sihem et al. proposed two clustering algorithms, k-means cluster based energy efficient routing algorithm (KEER) and its enhanced version (EKEER) for mobile UWSNs [11]. They use the k-means technique for the selection of initial cluster-heads and then replace each one with the sensor node that has the highest remaining energy in the next round, thereby increasing the network’s lifetime. However, KEER and EKEER cannot maintain high network performances because the sensor nodes may lose link connections during data delivery when the cluster-heads fail. The link holes cannot be recovered until new cluster-heads are selected at the beginning of the next round.

Non-clustered tree topology-based networks have been studied as an alternate approach for data gathering [13,14,15,16,17,18], where long communication links between cluster-heads do not exist. In [13], the concept of SON was employed to initially construct tree topological UWSNs. The initial network was built in an autonomous way by flooding a control packet, which departed from a sink node, throughout the network. However, the network topology was maintained without adapting the changes in the situation afterwards. Thus, the network connectivity becomes worse when a sensor node is newly added or lost. The US Navy’s Seaweb also employed a multi-hop tree topological network [14,15,16,17]. Unlike the early stages where Seaweb relied exclusively on users for manually configuring all of the communication links, the recent version of Seaweb allows the sensor nodes to autonomously establish the network based on the breadth-first search algorithm without the central control. However, both Seaweb and the scheme in [13] (referred to as $S_{13}$ hereafter) rely on network reorganization that happens periodically or in an event-triggered way to deal with link holes caused by the addition and loss of nodes. To maintain high network connectivity, the reorganization should be done frequently, which causes the overall energy consumption to increase considerably. Recently, underwater routing protocols [19] consider self-organizing concept to cope with the poor underwater conditions. An efficient hop-by-hop dynamic addressing based protocol named H2-DAB [18] has both network topology discovery and maintenance parts. The H2-DAB also discovers an initial network based on the flooding of a control packet like $S_{13}$ and handles topological changes caused by link holes. For delivering data to a sink, a sensor node first obtains the hop-count of its neighbors, i.e., the number of hops required to reach the sink. Then, the sensor node selects the node with the smallest hop-count as a relay. This hop-by-hop forwarding continues until the data reaches the sink. By reconfiguring a link as required, a sensor node is able to handle the loss of neighbors adaptively during data forwarding. However, the H2-DAB also requires network reorganization to enable failed nodes in hearing control packets for network initialization or to aid newly added nodes in joining the network without causing extra delays. In addition, the H2-DAB has a large signaling overhead associated with the acquisition of neighbors’ hop-count. Moreover, installing multiple sinks over a wide area is very expensive. In a pressure sensor based reliable routing protocol, named PSBR [20], sensor nodes exchange information of depth and residual energy with their neighbors. Then they calculate each neighbor’s link quality and select the best one as their relay. Since the information exchange repeats periodically, newly added or lost nodes can join or leave the network naturally depending on the period. The period should be adequate for trade-off between throughput and energy consumption.

In [21], a protocol for non-clustered and non-tree topological networks, called DIVE, was proposed for ID assignment and topology discovery. Nodes can obtain a globally unique ID and share topology information in a self-adaptive and distributed manner. However, DIVE does not consider the network maintenance.

Thus, designing a network that can be controlled in simpler way without causing heavy signaling or long delays is highly desired. This paper proposes architecture for a Self-Organized Underwater wireless sensor NETwork (SOUNET), which addresses issues of design complexity and network recovering delay. In the proposed scheme, after constructing an initial tree-topological network through the flooding of a control packet as in $S_{13}$ and H2-DAB, the sensor nodes consistently update a neighbor table, which contains information about neighbors such as their identification, hierarchical level or hop count, and the dead-end status indicator based on the packets heard from the neighbors. When a link hole occurs due to a lost or dead node, the child node reconfigures the link based on the local information acquired from the neighbor table. Such a scheme without additional signaling for finding new links not only saves energy consumption but also reduces delays for repairing networks. Moreover, unlike the previous works where network joining takes place only at the beginning of the network reorganization, in the proposed scheme newly added sensor nodes can immediately join the network by selecting a parent node whose packet is heard first. Additionally, SOUNET addresses other important issues such as *node-isolation* and *closed loops* [22,23,24]. SOUNET can resolve these issues by letting the isolated nodes sleep and by eliminating the loops through simple packet exchanges and local knowledge. Computer simulation results show that SOUNET outperforms other conventional schemes in terms of network connectivity, packet delivery ratio (PDR), and energy consumption over the whole network. In addition to computer simulation, we performed a lake experiment to verify some basic functions of SOUNET at the Gyeongcheon Lake in Korea.

This paper is organized as follows: in Section 2, we provide the system description. The subsequent section, Section 3, describes the problem statement. In Section 4, we present the detailed description of SOUNET. Section 5 and Section 6 discuss the simulation and experimental results, respectively. Finally, we conclude the paper in Section 7.

## 2. System Description

**Figure 1:**
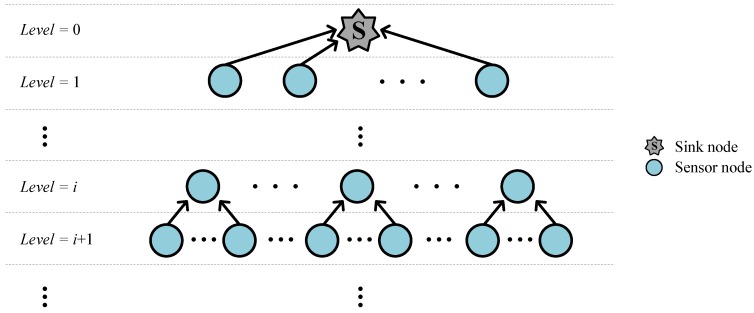
Hierarchical network topology of SOUNET.

We consider a typical data-gathering sensor network where one sink gathers environmental data from multiple normal nodes that are randomly distributed. We assume that all nodes have the same transmission coverage. From the aspect of network topology, the network is tree-shaped and the sink is located at the top, as shown in Figure 1. The *hierarchical level* denoted by an integer starting from 0 (highest level) for the sink is incrementally assigned to each level. The connection is made only between adjacent levels *i* and *i* +1, where the nodes at *i* and *i* + 1 are called the *parent* and *child* of each other, respectively. The nodes associated with the data flow from the lowest level up to the sink compose a *branch*, where the bottom node is called a *leaf* and nodes at higher and lower levels are called *ancestors* and *descendants*, respectively. *Neighbors* are defined as those within the communication range, including parent, child, and possibly several nodes of other branches. Each node keeps the information about neighbors in the so-called *neighbor table.* For medium access control, we employ ALOHA protocol [25], which is well suited to the underwater channel due to its simplicity and insensitivity to large propagation delays [26]. In addition, since ALOHA does not require time synchronization between nodes, it is assumed that all nodes are not synchronized and are homogeneous in terms of capability and physical specification.

In this paper, a node is called a *dead end* when it has no more valid parents other than the current parent, if any. Moreover, a node is called *isolated* when it is completely disconnected from any other nodes. A node becomes isolated necessarily through the state of a dead end. Similarly, a group of nodes is called *isolated* when the group has no connection leading to the sink, even though the nodes inside the group are connected.

## 3. Problem Statement

To improve the network’s sustainability, repairing the link holes caused by the loss of nodes should be at the earliest. For instance, a child node losing connection must find a new parent or the lost nodes should be replaced by new ones as soon as possible. During such recovery, the following issues arise.

### 3.1. Detour and Isolation

**Figure 2:**
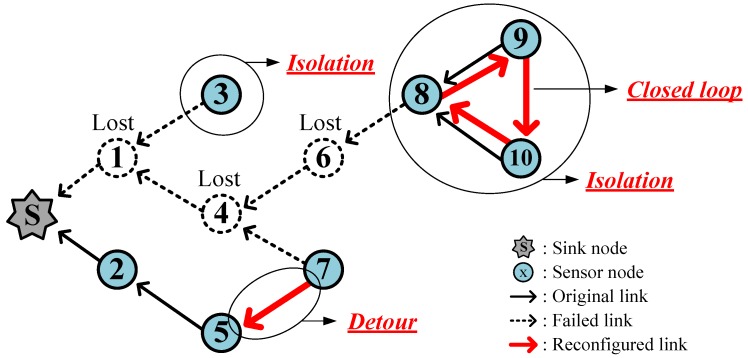
Example of detour and isolation due to lost nodes.

Figure 2 illustrates two types of incidents that may occur when sensor nodes are lost. Each number inside the circles identifies a node. Assume that the nodes 1, 4, and 6 are lost.

Then the node 7, which was the child of node 4, builds a new communication link (detour) with node 5 so that the data gathering can continue. Meanwhile, node 3, which was the child of node 1, fails to find an alternative parent and loses all connections (isolation), and the nodes 8–10 also become disconnected from the sink, and thus, form an isolated group. Relevant nodes appropriately recognize such isolations so that they can enter sleep mode to save energy until they are repaired. To do this, the isolated nodes broadcast a control packet named *ISO* to announce the isolation to all neighbors.

### 3.2. Closed Loops

In the process of detouring, shortsighted nodes (i.e., nodes 8–10 in Figure 2) may form a *closed loop* where data goes round infinitely and does not reach the sink. Such a closed loop should be recognized and fixed in an efficient way.

### 3.3. Network Joining Delay

It is essential for delay-critical data to reach the sink in time. If newly added nodes stay disconnected for a long time, the associated data may expire and ends up discarded. Thus, quickly reconfiguring the network by acquiring local information from the surroundings is highly required.

## 4. Construction and Operation of SOUNET

SOUNET constitutes of two phases: *network initialization* and *network maintenance*. In the network initialization phase, sensor nodes start network topology discovery via packet flooding initiated by a sink. Next, in the network maintenance phase, the sensor nodes update the links by referring to the neighbor table while forwarding data to the sink. The neighbor table contains three parameters of neighbors: an *identifier* (*ID*), a *hierarchical level* (*LV*) and an *indicator that represents whether the neighbor is a dead end or not* (*DE*).

### 4.1. Phase 1: Network Initialization

After node deployment, the sink begins the network initialization by flooding a control packet named *HELLO*, which contains the sender’s *ID* and *LV*. On receiving the *HELLO*, sensor nodes identify their parent and hierarchical level. The nodes ignore the *HELLO*s received from the same or lower level. The *HELLO* flooding continues until the *HELLO* reaches leaf nodes. Naturally, the revision of the *HELLO* with new senders’ *ID*s and *LV*s occurs at every flooding. Due to packet collisions, however, some sensor nodes may not receive the *HELLO* successfully. Such sensor nodes join the network later by overhearing a *DATA* packet in the next phase.

### 4.2. Phase 2: Network Maintenance

In this phase, during the data transfer between the sensor nodes to the sink, updates are performed on the communication links according to the topological changes such as node additions and losses.

#### 4.2.1. Data Transfer

On acquiring data, the sensor node sends *DATA* packets to the parent. Then the parent replies with an acknowledge packet, *ACK*. Such an exchange of *DATA* and *ACK* continues along the branch until the *DATA* reaches the sink. During data transfer, if necessary, the associated sensor nodes update the neighbor table.

#### 4.2.2. Link Hole Recovery

The link hole occurs when a sensor node is lost due to battery discharge or physical damage such that the branches connected to the lost node are disconnected. Since the link hole causes data loss, it is essential to recover the link hole by creating a detour or by replacing the lost node immediately with a new one. However, the problem lies in the way the sensor nodes recognize the occurrence of link holes without centralized notification. In SOUNET, the sensor nodes count the number of failures in *ACK* reception, $n_{\mathrm{ack}\_\mathrm{fail}}$, from the parent and if the value reaches the threshold, $N_{\mathrm{MAX}\_\mathrm{ACK}\_\mathrm{FAIL}}$, then they may regard that parent node as a link hole. The $n_{\mathrm{ack}\_\mathrm{fail}}$ becomes zero when an *ACK* or any type of packet is received, indicating that the parent is alive.

**Figure 3:**
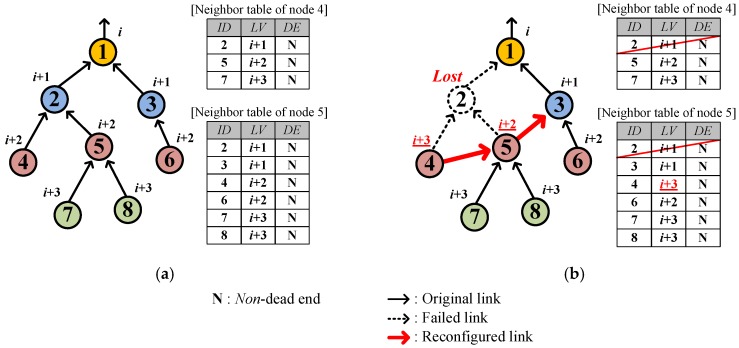
Example of link hole recovery: (**a**) Before link hole occurrence; (**b**) After link hole recovery.

On recognizing a link hole, a sensor node starts the link hole recovery by deleting the current parent and searching for a new parent in the neighbor table. The sensor node randomly selects the new parent node from *among the highest-leveled neighbors who are neither dead ends nor child nodes*. Regarding whether the neighbor is a dead end or not, the node easily obtains the *DE* status of neighbors without extra signaling by overhearing *DATA* from neighbors since the *DATA* contains the sender’s *DE* in the packet header. Figure 3 shows an example of link hole recovery where eight nodes (from 1 to 8) are located in levels from *i* to *i* + 3, and the node 2 is lost (dotted circle in Figure 3b) and accordingly the neighbor tables of associated nodes 4 and 5 are updated. Nodes 4 and 5 end up detouring via new parents 5 and 3, respectively.

**Figure 4:**
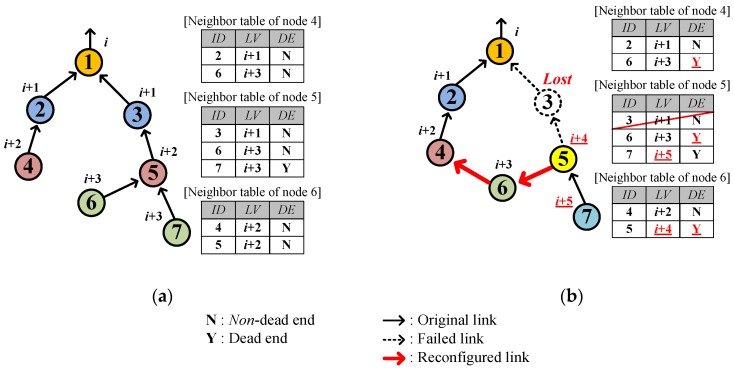
Additional example of link hole recovery: (**a**) Before link hole occurrence; (**b**) After link hole recovery.

If no valid parent candidate exists, the sensor nodes inevitably select a new parent from *among their child nodes*. Figure 4 shows such an example where node 3 is lost and then node 5 selects node 6 as a new parent because node 7 is a dead end. In this case, node 5 also becomes a dead end because no alternative candidate for parent exists. Node 6 realizes that it is selected back as a parent by the current parent, node 5, on receiving a *DATA* destined for itself from node 5. In addition, node 6 learns that node 5 became a dead end by reading the *DE* field of the *DATA* sent by node 5 and it also becomes a dead end. After that, node 6 updates the neighbor table accordingly and selects node 4 as the new parent. Later, nodes 4 and 2 also become dead ends.

#### 4.2.3. Isolation Recognition

Link holes may cause isolation of the associated descendants. Suppose that node 2 is additionally lost in Figure 4b. Then the group of nodes from 4 to 7 becomes isolated because the node 1 is located beyond their transmission coverage. Without recognizing the isolation, the nodes inside isolated group may keep wasting energy in transmitting data. Thus, it is necessary to recognize the isolation and enter sleep mode shortly for saving energy. In SOUNET, a dead end recognizes isolation under one of the following conditions:

- Receiving *ISO* from the parent.
- Receiving *DATA* back from the parent who becomes a dead end.
- $n_{\mathrm{ack}\_\mathrm{fail}}$ reaches $N_{\mathrm{MAX}\_\mathrm{ACK}\_\mathrm{FAIL}}$.

**Figure 5:**
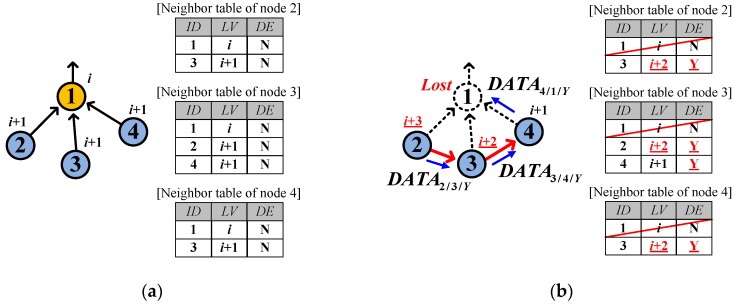

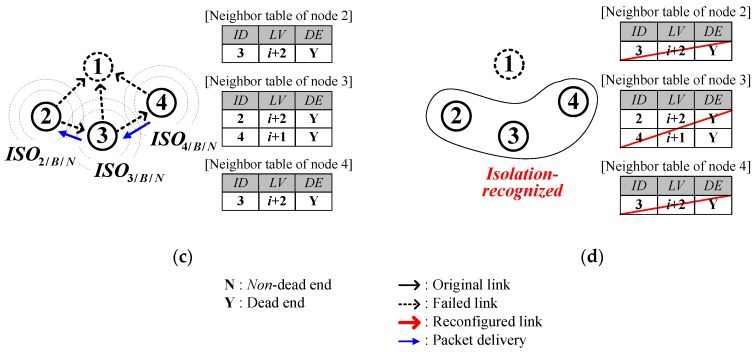
Example of isolation recognition: (**a**) Before link hole occurrence; (**b**) Link hole occurrence and searching for new parents; (**c**) Recognizing isolation; (**d**) Sleeping in isolation.

After recognizing the isolation, the isolated node immediately broadcasts an *ISO* and enters sleep mode until it finds a valid parent. Figure 5 shows an example of isolation recognition where nodes from 2 to 4 have a common parent, node 1 (Figure 5a). In case node 1 is lost, the link hole creates the isolated group that includes nodes 2, 3, and 4 (Figure 5b). Assume that the link hole is recognized by the order of nodes 2, 3, and 4. Then, node 2 chooses node 3 as the new parent and becomes a dead end due to absence of an alternative parent. Node 3 figures out what happened in node 2 by receiving *DATA*_2/3/Y_, where the subscript 2/3/Y represents the sender ID, receiver ID and sender’s *DE*, respectively. After that, node 3 chooses node 4 as the new parent and becomes a dead end too. In the same way, node 4 figures out node 3’s situation. Recognizing that no valid parent exists, node 4 broadcasts an *ISO*_4/B/N_, where the subscript 4/B/N denotes the sender ID, broadcasting mode and flag for loop existence (explained later), respectively (Figure 5c). On receiving the *ISO* from node 4, node 3 recognizes that it is also isolated. In the same way, node 2 also recognizes the isolation (Figure 5d).

Meanwhile, if node 4 can reach node 2 in the previous example of Figure 5, the isolated group of nodes 2, 3, and 4 may form a loop, causing data to circulate infinitely. With the limited information contained in *DATA* such as sender ID, receiver ID and sender’s *DE,* which are described as the subscripts in the previous example, the nodes of isolated group will not be able to recognize the loop. To solve this problem, some additional information needs to be contained in *DATA* such as the source node ID at which the *DATA* was originated (source ID), the number of relays the packet has been through ($n_{\mathrm{relay}}$), and the node ID that detected the existence of a loop (*LDID*). A sensor node detects a closed loop under one of the following conditions:

- Receiving a *DATA* of which source ID is its own.
- Receiving a *DATA* of which $n_{\mathrm{relay}}$ value reaches the threshold, $N_{\mathrm{MAX}\_\mathrm{RELAY}}$.

**Figure 6:**
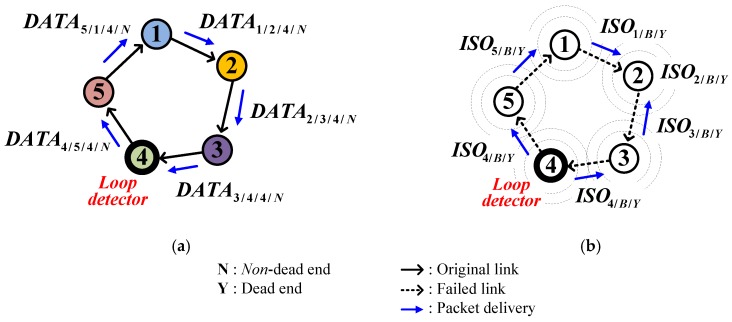
Way of handling a loop of an isolated group: (**a**) Sharing the information that a loop is detected; (**b**) Sharing the information that the loop is isolated.

Upon recognizing the loop, the sensor node called loop detector (*LD*) puts its own ID into the *LDID* field and continues to relay the *DATA*. Then subsequent nodes on the loop recognize the loop by reading the *LDID.* Finally, the *LD* receives back the *DATA* with its own *LDID* and then broadcasts the *ISO* containing the loop flag set to ‘Y’ and enters a sleep mode. After receiving the *ISO*, the remaining nodes on the loop sequentially follow what the *LD* did by broadcasting the *ISO* and entering a sleep mode. Figure 6 shows an example to handle the loop of an isolated group. Assuming that node 4 is an *LD*, it transmits *DATA*_4/5/4/N_ where the subscript 4/5/4/N represents the sender ID, receiver ID, *LDID* and sender’s *DE*, respectively (Figure 6a). Upon receiving back the *DATA,* node 4 broadcasts an *ISO* and sleeps, and the remaining nodes sequentially follow the node 4 (Figure 6b).

In case there is a failure in receiving *DATA* due to collisions, which may occasionally happen, especially when the network is crowded, the loop detection is difficult. In the proposed SOUNET, the value of *LV* makes the detection of loop easier. *LV* is updated whenever a packet is overheard from the neighbors even though *DATA* is missed and if *LV* keeps increasing up to a certain threshold, $N_{\mathrm{MAX}\_\mathrm{LEVEL}}$, it is assumed that the loop is formed.

#### 4.2.4. Temporary Closed Loop and Its Cure

**Figure 7:**
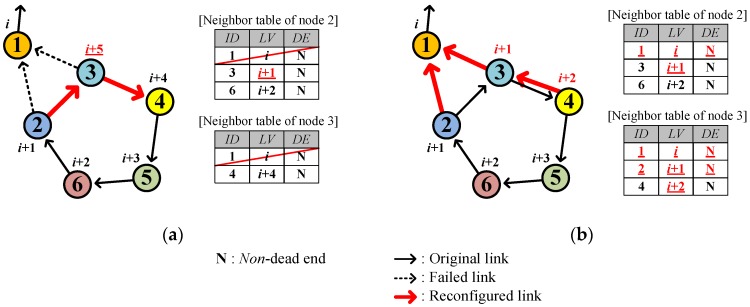
Example of a temporary loop: (**a**) Forming; (**b**) Eliminating.

Since SOUNET relies on local information (neighbor table), which requires time to be updated, a temporal discrepancy between the neighbor tables occurs. Such a discrepancy may temporarily create a closed loop. However, it is easy to cure a *temporary closed loop* through the neighbor table update. Figure 7 illustrates the formation and curing of a temporary closed loop.

Assume that the node 3 accidently loses connection to the current parent node 1, which is alive. Then, not recognizing the existence of node 2, node 3 selects node 4 as the new parent (Figure 7a) and accordingly the *LV* of node 3 is changed from *i* + 1 to *i* + 5. Meanwhile, node 2 also accidently loses connection to node 1 before knowing the *LV* of node 3 is updated and selects node 3 as the parent based on the neighbor table which shows that the *LV* of node 3 is still *i* + 1. Consequently, a loop consisting of nodes from 2 to 6 is temporarily formed. However, the loop is easily eliminated if either node 2 or node 3 overhears a packet from node 1 anytime afterwards (Figure 7b).

#### 4.2.5. Fast Network Joining

Similar to the initial network joining in the initialization phase, during the network maintenance phase newly added sensor nodes can also join the network immediately by selecting a parent whose packet (*HELLO* or *DATA*) is overheard first. Even though the first parent may not be the best one, a better one (that has a higher LV) can replace it later through the continuous update of the neighbor table.

## 5. Simulation Results

An event-driven network simulator is used which was developed in our laboratory based on MATLAB (R2013a) and has been used to evaluate many schemes of medium access control and networking for underwater communication [27,28,29,30,31]. We compare the proposed SOUNET with Seaweb and $S_{13}$. To the best of our knowledge, Seaweb and $S_{13}$ are only ones that employ self-organization techniques under the same conditions as ours, i.e., one sink and multiple nodes are randomly distributed, and they all have the same transmission coverage.

### 5.1. Simulation Model

**Figure 8:**
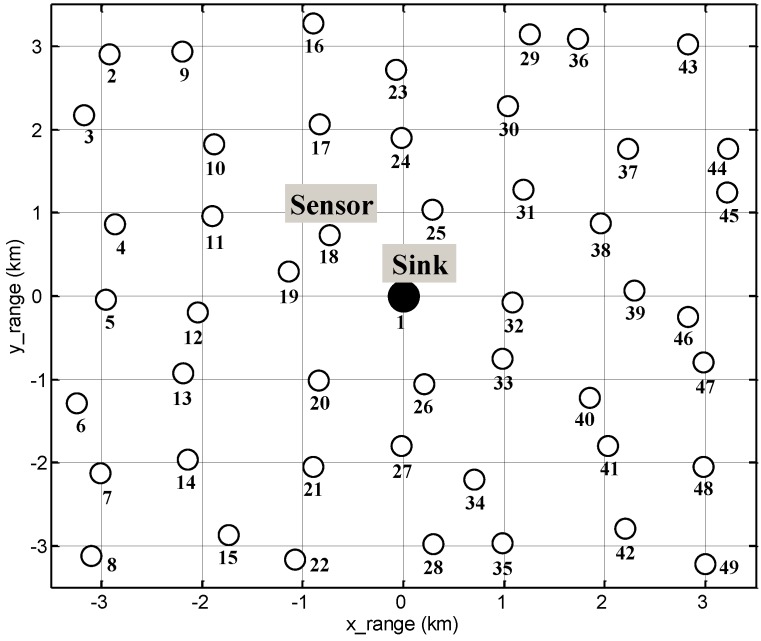
Node deployment.

Figure 8 shows the node deployment, where total 48 nodes (circles) are located around each grid point with 10% variation in the spacing and a sink (filled circle) at the center of the network. Each transmission range could be different in real world applications, but we assumed that all of the nodes have the same transmission range. We believe that the differences are inevitable errors since it is too complicated and difficult to solve but can be avoided if the nodes are placed accordingly such that the differences do not occur. The traffic generated by a node follows the Poisson arrival process with a rate of λ. In cases when multiple *DATA* packets need to be transmitted, the nodes prioritize the relayed data from the neighbors over their own data. In addition, we assumed that the nodes operate in half-duplexing mode [32] and the packet loss is considered to occur only through collisions.

Table 1 summarizes the system parameters used in the simulation. For the values of the data rate and the power consumed for transmission and reception, we refer to the commercial Teledyne Benthos ATM-885 underwater modem [33].

### 5.2. Simulation Scenario

**Figure 9:**
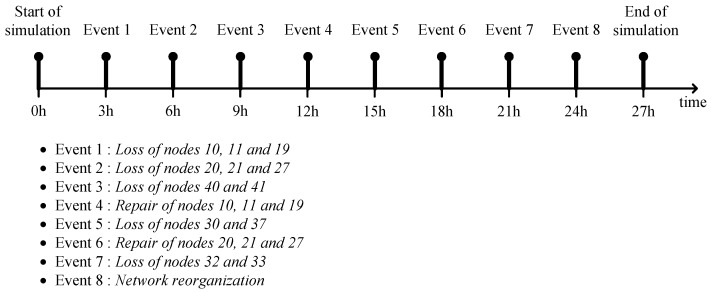
Simulation scenario.

Figure 9 shows a simulation scenario on the timeline where the events of loss or addition of nodes occur every 3 hours during the whole simulation time of 27 h. We assume that the newly added nodes are located at the same position as the lost ones. At 24 h, the sink broadcasts a *HELLO* to reorganize the network, starting all over again.

### 5.3. Analysis of Results

**Figure 10:**
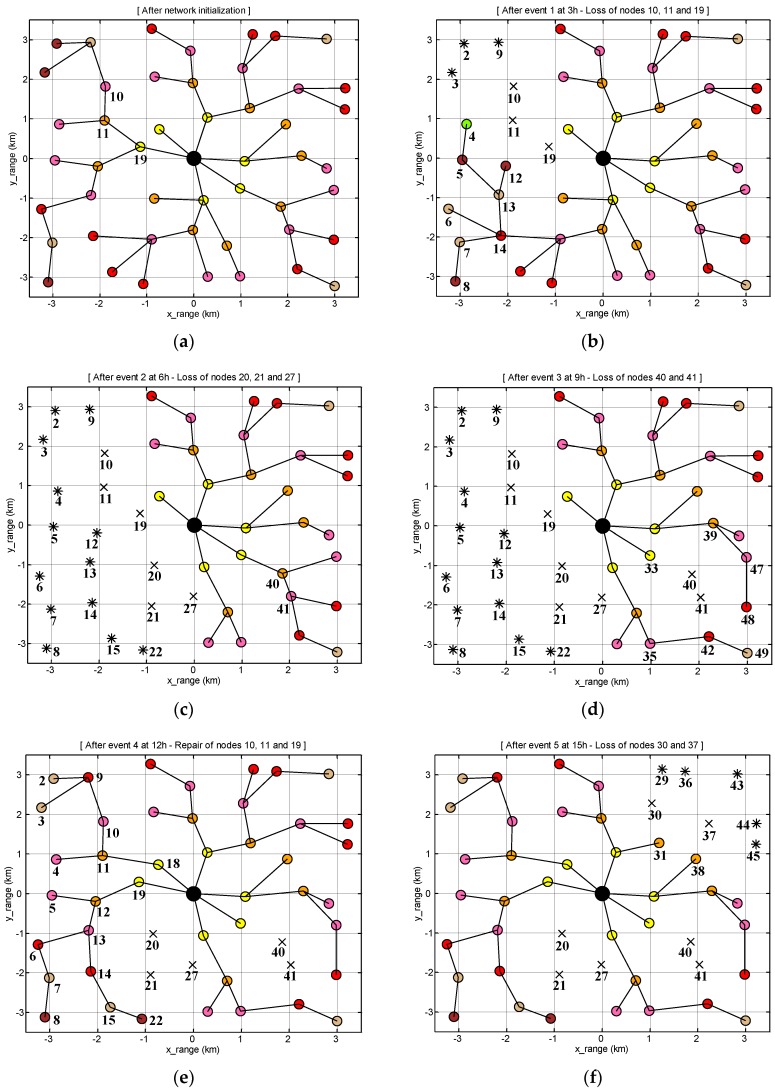

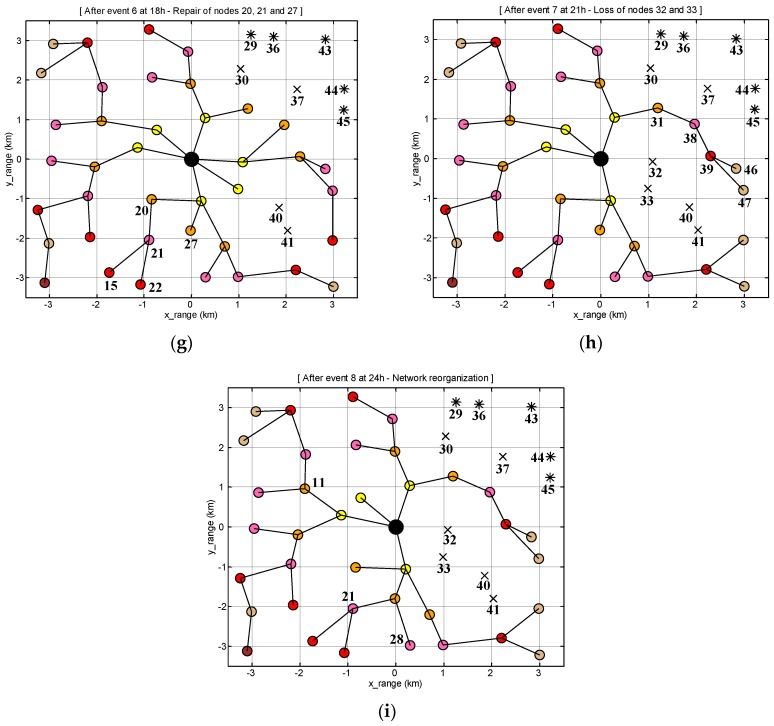
Various network stages of SOUNET: (**a**) After initialization; (**b**) After the event 1; (**c**) After the event 2; (**d**) After the event 3; (**e**) After the event 4; (**f**) After the event 5; (**g**) After the event 6; (**h**) After the event 7; (**i**) After reorganization.

Figure 10 shows how the connections between the nodes change according to the scheduled scenario of Figure 9. Initially, the nodes organize a tree-topological network (Figure 10a). At 3 h (Figure 10b), the nodes 10, 11, and 19 are lost as marked by ‘×’ and thus their descendants (nodes 2–9 and 12–14) become temporarily detached from the network. Then, the nodes 4–8 and 12–14 find detouring paths while the nodes 2, 3, and 9 fail in detouring and recognize isolation as marked by ‘∗’. At 6 h (Figure 10c), the nodes 20, 21, and 27 are additionally lost, and thus the corresponding descendants (nodes 4–8, 12–15, and 22) are isolated. At 9 h (Figure 10d), the nodes 40 and 41 are newly lost, but this time, the descendants (nodes 47 and 48) all find new parents and successfully detour. At 12 h (Figure 10e), the nodes 10, 11, and 19 are repaired (sometimes they can be replaced by new ones) and the remaining isolated nodes are all connected to the network again. At 15 h (Figure 10f), the nodes 30 and 37 are newly lost and consequently the nodes 29, 36, and 43–45 are isolated. At 18 h (Figure 10g), the nodes 20, 21, and 27 are repaired and the nodes 15 and 22 adaptively reconfigure their connection in such a way that their hierarchical levels become higher. At 21 h (Figure 10h), the nodes 32 and 33 are newly lost and their corresponding descendants nodes 38, 39, 46, and 47 detour through the node 31. At 24 h (Figure 10i), the network is reorganized from the start.

Without repairing or battery recharging, the nodes 30, 32, 33, 37, 40, and 41 are still lost and thus the associated descendants, nodes 29, 36, and 43–45 remain isolated. On the other hand, nodes 11, 21, and 28 find new parent during the reorganization. From the above scenario, we observed that the proposed self-organizing methodology works well to adapt the network variations such as node loss or node addition.

#### 5.3.1. Network Connectivity

**Figure 11:**
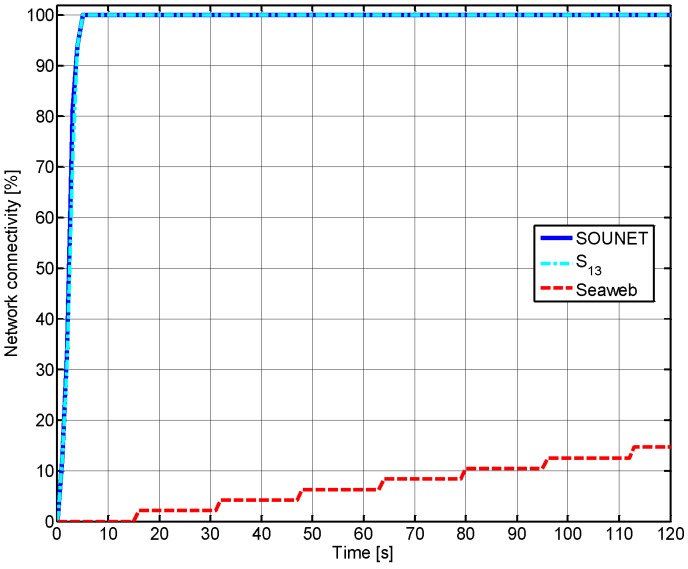
Variation of network connectivity at the beginning of simulation (0 s to 120 s).

The network connectivity (*C*) is defined by:

(1) $$C=\frac{N_{\mathrm{connect}}}{N_{\mathrm{awake}}},$$

where $N_{\mathrm{awake}}$ and $N_{\mathrm{connect}}$ are the number of nodes that are awake, which means alive but not sleeping in isolation and the number of nodes that are connected to the sink, respectively. Obviously, the network with a larger *C* can cover a wider area. Figure 11 shows how the connectivity changes at the beginning of node deployment from 0 s to 120 s. At the beginning, SOUNET and $S_{13}$ achieve 100% connectivity very quickly within several seconds while Seaweb requires much more time. The Seaweb’s connectivity reaches only 15% until 120 s and ends up at 100% after 15 min. The quick achievement of 100% connectivity results from the *HELLO* packet flooding in the network initialization while the breadth-first search algorithm [34] is used in Seaweb.

**Figure 12:**
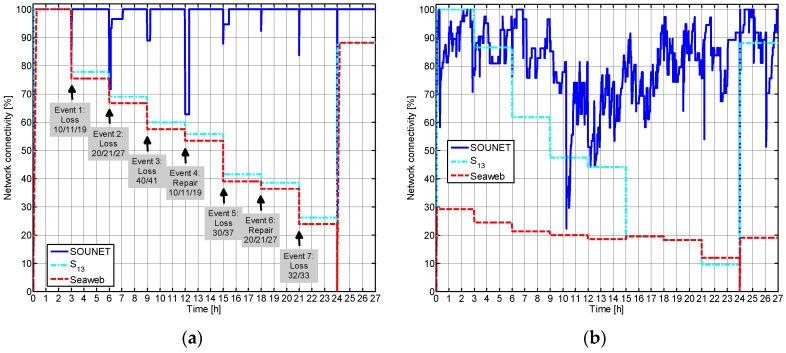
Variation of network connectivity during the entire simulation time (0 h to 27 h): (**a**) Considering only packet collisions; (**b**) Considering both packet collisions and bit errors.

Figure 12 shows how the connectivity changes during the whole simulation time in which the incidents occur every three hours as planned from 0 h to 27 h, respectively. As the incident of node loss occurs at 3 h, 6 h, 9 h, 15 h, and 21 h, the connectivity sharply decreases since the group of nodes associated with the lost nodes is disconnected at once. However, since the proposed scheme allows the disconnected nodes to detour through the new parents, the connectivity is recovered shortly while in the other schemes the connectivity remains unrecovered. Although the formation of closed-loops are possible during the detour process, the nodes can detect and eliminate them. In our scenario, a group of nodes 2, 3, and 9 and another group of nodes 4–8, 12–15, and 22 could form such closed loops after event 1 and 2, respectively.

It is shown that the first group of nodes 2, 3, and 9 can resolve the loop immediately after the event occurs while the second group of nodes 4–8, 12–15, and 22 may take more time to resolve it. Normally, as a group of isolated nodes is bigger, it takes more time to recognize isolated or closed loops. The node addition at 12 h and 18 h also temporally decreases the connectivity since the denominator, the number of nodes that are awake, $N_{\mathrm{awake}}$ increases. However, in the proposed scheme the added nodes quickly repair the damaged connectivity by building a new connection with the isolated nodes or by rebuilding the existing connection so that the paths to the sink become shorter. Thus, the recovery of connection is very quick. Meanwhile, both Seaweb and $S_{13}$ recover the connectivity only when the network is reorganized at 24 h, not being capable of dealing with the incidents of loss or addition of nodes. Even at the network reorganization, the recovery of Seaweb and $S_{13}$ remains below 90%. When we add bit errors that reflects the harsh underwater channel condition, as well as packet collisions (Figure 12b), SOUNET’s connectivity fluctuates with time because nodes continuously change their parent node. On the other hand, performances of $S_{13}$ and Seaweb decreases considerably. For Seaweb, in particular, the initial connectivity at 0h and 24h suffers a significantly large drop (compared with Figure 12a) since many control packets for network initialization are lost. SOUNET clearly outperforms the others and it highlights SOUNET’s robustness to the unreliable and peculiar characteristics of underwater channel.

#### 5.3.2. Packet Delivery Ratio

**Figure 13:**
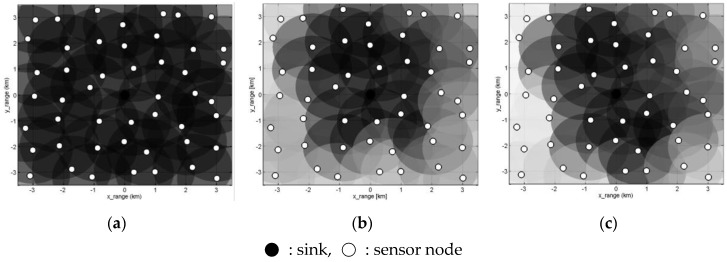
Visualization of $\rho_{i}$ over the whole area: (**a**) SOUNET; (**b**) $S_{13}$; (**c**) Seaweb.

The packet delivery ratio (PDR) of a node *i* is defined by:

(2) $$\rho_{i}=\frac{D_{\mathrm{delivered}}^{i}}{D_{\mathrm{sent}}^{i}},$$

where $D_{\mathrm{sent}}^{i}$ and $D_{\mathrm{delivered}}^{i}$ are the numbers of *DATA* packets sent by node *i* and numbers of *DATA* packets delivered to the sink, respectively. Figure 13 visualizes $\rho_{i}$ by coloring in a way that the circles denoting the coverage of individual nodes become darker with higher PDR. It is shown that SOUNET has darker $\rho_{i}$ over the whole area while Seaweb and $S_{13}$ have dark regions only near the sink. This is because descendants of the lost nodes (normally located at the outer region of the network) keep sending *DATA* packets even though their lost parents are not able to relay them toward the sink. Rather, the packets cause more collisions to neighbors. On the other hand, SOUNET not only maintains high connectivity even at the edge of the network but also reduces unnecessary DATA transmission by forcing the isolated nodes to sleep.

**Figure 14:**
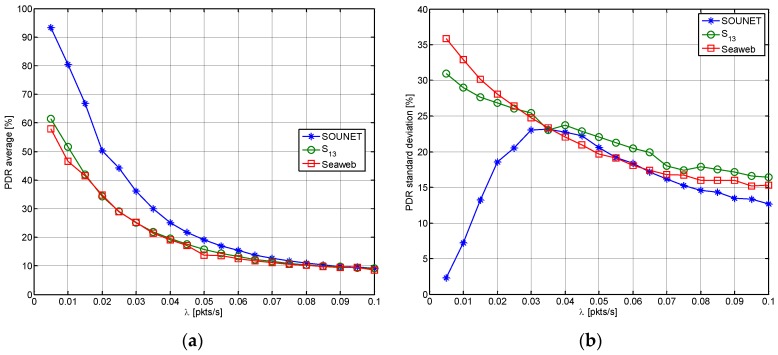
The average and SD of $\rho_{i}$: (**a**) $\mu_{\rho }$; (**b**) $\sigma_{\rho }$.

Figure 14 shows the average and standard deviation (SD) of $\rho_{i}$, which are denoted by $\mu_{\rho }$ and $\sigma_{\rho }$, respectively, with varying λ. For each value of λ, the corresponding values of $\mu_{\rho }$ and $\sigma_{\rho }$ are obtained by running the whole simulation scenario of Figure 9. SOUNET shows better performance in terms of both $\mu_{\rho }$ and $\sigma_{\rho }$. That is, larger number of packets are successfully delivered to the sink and packets are more evenly collected from the entire network. That is because the SOUNET maintains high connectivity by detouring even when unexpected link holes occur. As λ increases, the funneling effect [35] dominantly appears and thus PDR averages of the all schemes become undistinguishable near 10%. On the other hand, $\sigma_{\rho }$ of SOUNET, which is quite small under low traffic conditions, becomes larger as λ increases since some nodes selectively have larger collisions compared to others and then becomes smaller again as λ increases further since more nodes commonly experience the collisions.

#### 5.3.3. Energy Consumption

**Figure 15:**
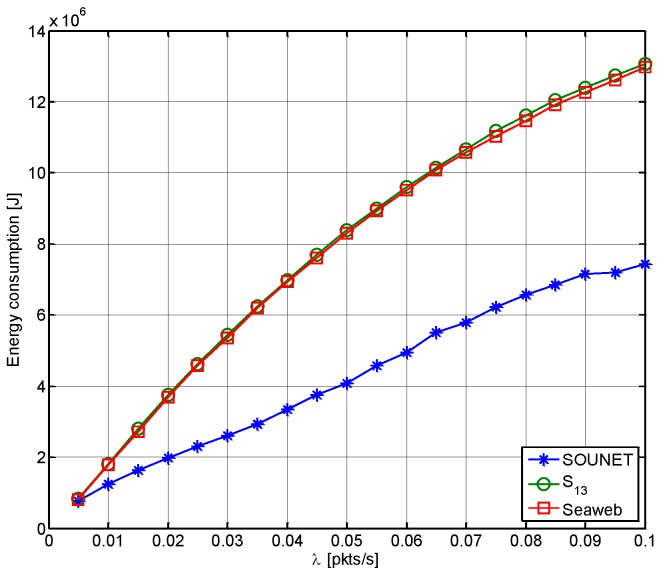
Energy consumption.

Figure 15 shows the total energy consumption for transmission and reception of all kinds of packets. The energy consumed during sleeping and CPU processing is ignored. As λ increases, the energy consumption increases since more *DATA* packets attempt transmission. However, comparing to Seaweb and $S_{13}$ where the isolated nodes keep trying to send *DATA* and end up exhausting battery, SOUNET can save considerable energy even at high traffic loads by letting the nodes recognize any possible isolation by themselves and sleep to save energy.

#### 5.3.4. Signaling Overhead

*HELLO* packet flooding is a common method used for initializing a network and SOUNET and $S_{13}$ apply this method. As the network size increases, the number of *HELLO* packets transmitted by nodes also grows, thus increasing the necessary signaling overhead. In our simulation, 49 *HELLO* packets are transmitted during initialization (in the case of SOUNET and $S_{13}$). On the other hand, the other comparing scheme, Seaweb, uses the breadth-first search algorithm instead of the *HELLO* flooding. This leads to higher number of signaling overhead; for example, in total about 900 packets were obtained from our simulation. During the maintenance phase, SOUNET adapts to the varying environment with the help of the overhearing packets. Therefore, no additional signaling is required and no large overhead is incurred during maintenance. $S_{13}$ and Seaweb do not have maintenance phase.

## 6. Experimental Results

**Figure 16:**
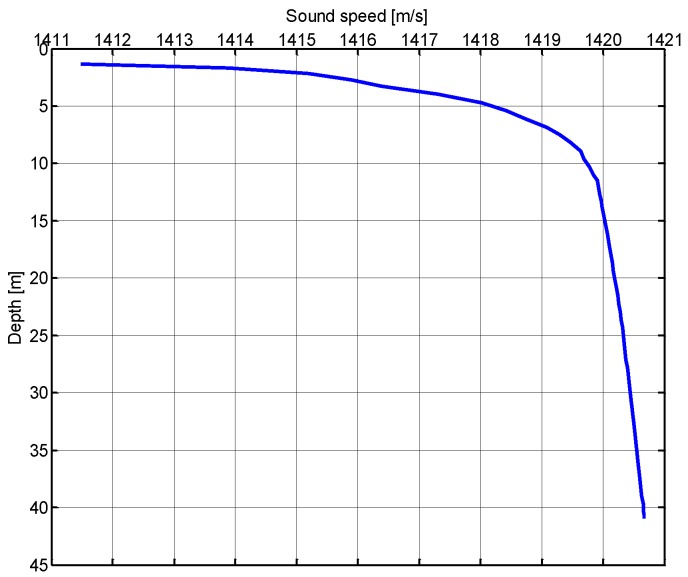
Sound speed profile.

**Figure 17:**
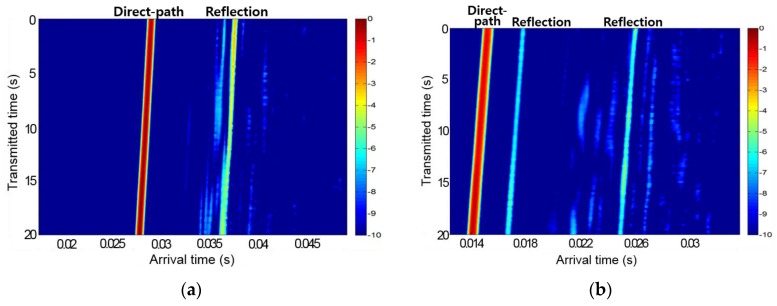
Channel impulse response: (**a**) Channel 1; (**b**) Channel 2.

The underwater experiment on SOUNET was performed on 27 May, 2015 at the Gyeongcheon Reservoir in Mungyeong City, Korea. Figure 16 and Figure 17 show the sound speed profile and channel impulse responses we measured at the Gyeongcheon Reservoir. The depth of water measured about 10–40 m and bottom of the reservoir was mud.

**Figure 18:**
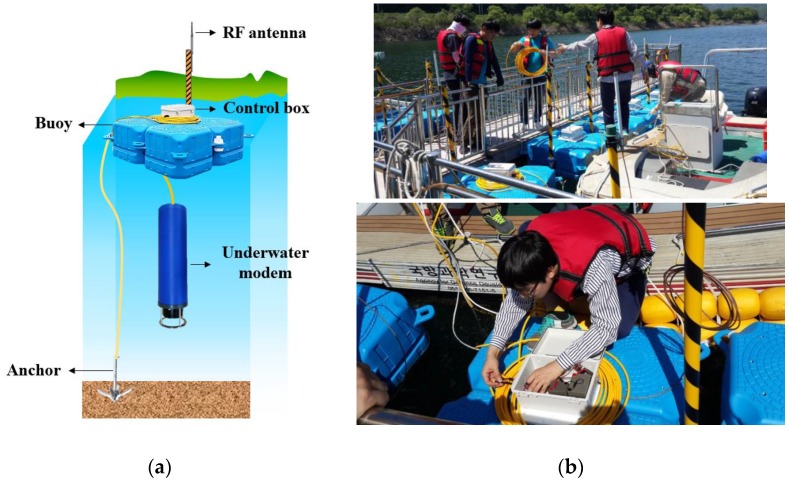
Field experiment: (**a**) Node components; (**b**) Node installation.

Due to limited budget, only seven nodes, including one sink, were deployed, each consisting of an underwater modem, an RF antenna, a control box, a buoy, and an anchor as shown in Figure 18a. The control box contains a DSP board implemented by ATmega2560 to control the underwater modems. In addition, the DSP board reports every packet exchange between the nodes underwater to an offshore central station through RF communication. Then the central station implemented by a notebook computer shows the events through the Windows GUI program we developed. For the underwater modem, the model ATM-885 (9–14 kHz) manufactured by Teledyne Benthos was used. The buoy was used to carry the control box on the water surface as well as to denote the node location. Anchors were used to hold the nodes at a static position.

**Figure 19:**
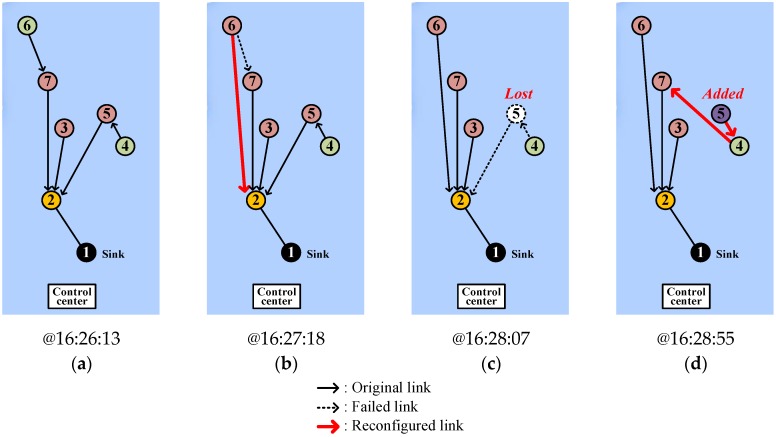
Various network stages of SOUNET during the experiment: (**a**) After initialization; (**b**) After change of node 6’s parent; (**c**) After node 5’s loss; (**d**) After node 5’s addition.

Figure 19 shows the changes of node connection in a self-organizing manner according to the events we scheduled, which the control station displays. Node 1 plays the part of sink. Initially, the connections are built up by means of *HELLO* flooding (Figure 19a). Since node 4 is blocked by an underwater mound and thus cannot hear from nodes 1, 2, and 3, it chooses node 5 as its parent. Node 6 that is connected to node 7 at the beginning updates the connection to node 2 upon overhearing node 2 to reduce the number of hops to the sink (Figure 19b). After that, we intentionally turned off node 5 to emulate a link hole between nodes 4 and 5 (Figure 19c). Soon after that, node 4 discovers the new parent, node 7 and retains the connection. Then we turned on node 5 again to emulate a node addition. The newly added node 5 temporarily tries to connect to node 4 (Figure 19d), but shortly recognizes a better possible connection and then tries to rebuild the connection. Finally, the network architecture returns to the beginning of Figure 19a.

## 7. Conclusions

We have proposed a new self-organized underwater sensor network, SOUNET, which addresses many practical issues caused by the unexpected loss or addition of sensor nodes. Unlike the previous works where manual reconfiguration or periodic network reorganization is required to handle such issues, SOUNET allows the nodes to recognize the loss or addition of neighbors and then to rebuild the connections or detour to the sink without any centralized control. Newly added nodes are also able to join the network immediately only by overhearing packets exchanged between neighbors. In addition, SOUNET resolves the troubles such as isolation and closed loop that may occur during network repairing in a self-healing manner. Moreover, by allowing the nodes that recognize isolation to sleep, we can considerably save energy. Simulation results showed that SOUNET outperforms other previous schemes in terms of network connectivity, PDR, and energy consumption. In future work we plan to perform additional underwater experiments for verifying the performance of SOUNET further and to study how to determine the existence of closed loops implicitly based on machine learning techniques, instead of explicit packet exchanges.

## Figures and Tables

**Table 1:** System parameters.

Parameter	Value
Grid spacing	1 km
Data rate	2400 bps
Propagation speed	1500 m/s
Transmission/Sensing range	1300 m
*HELLO*/*ACK*/*ISO* packet length	120 bits
*DATA* packet length	1200 bits
Tx mode power	20 W
Rx mode power	756 mW
$N_{\mathrm{MAX}\_\mathrm{ACK}\_\mathrm{FAIL}}$	3
$N_{\mathrm{MAX}\_\mathrm{RELAY}}$	10
$N_{\mathrm{MAX}\_\mathrm{LEVEL}}$	10
λ	0.001 packets/s
